# Lactobacillus for Vaginal Microflora Correction

**DOI:** 10.5195/cajgh.2014.171

**Published:** 2014-12-12

**Authors:** Saule Saduakhasova, Almagul Kushugulova, Gulnara Shakhabayeva, Samat Kozhakhmetov, Zhanagul Khasenbekova, Indira Tynybayeva, Talgat Nurgozhin, Zhaxybay Zhumadilov

**Affiliations:** Center for Life Sciences, Nazarbayev University, Astana, Kazakhstan

**Keywords:** vaginal microflora, lactobacillus, bacterial vaginosis, probiotics

## Abstract

**Introduction:**

Despite the significant progress made in prevention, diagnosis, and treatment, there is still a high rate of vaginal dysbiosis in Kazakh women. The use of antibiotics in the treatment of vaginal dysbiosis contributes to the elimination of pathogens as well as microflora, which can lead to a decrease in local immunity and more favorable conditions for infection spread. The most physiologically safe and promising method for the restoration of vaginal biocenosis is the use of probiotics administered by a vaginal route.

**Methods:**

We have allocated 64 of cultures of Lactobacillus from the vaginal epithelium of healthy women of reproductive age and women with diagnosed bacterial vaginosis (BV). Identification of cultures was performed by PCR analysis of 16S ribosomal RNA. Evaluation of biological significance was determined by the following criteria: high antagonistic activity against Candida albicans, Escherichia coli, Serratia marcescens, Proteus mirabilis, Klebsiella ozaenae, and Staphylococcus aureus; and production of hydrogen peroxide, resistance to antibiotics, adhesive activity. We studied the symbiotic relationship of selected biologically active of cultures to each other and received options for consortiums with properties of probiotics through co-cultivation.

**Results:**

Results of genotyping showed that the isolated lactobacilli belong to the seven species: L. fermentum, L. salivarius, L. gasseri, L. crispatus, L. jensenii, L. plantarum, and L. delbrueskii. L. fermentum, L. salivarius, L. gasseri, and L. jensenii occur in women with suspected BV. The highest percentage of occurrence in the vagina of healthy women was L. fermentum (28%). Most strains of lactobacilli possess high inhibitory activity for all test-strains, except Candida albicans (37.5%). 56% of studied cultures revealed high adhesion to human erythrocytes. All lactobacillus strains were resistant to metronidazole, 80% to kanamycin, 57% to vancomycin, and sensitivity to roxithromycin, amoxiclav, ampicillin was diagnosed in all strains. 50% of cultures showed a moderate sensitivity to gentamicin and cefazolin. In a study of peroxide-producing activity, 80% of the cultures exhibited peroxide-producing activity. As a result of screening, the 7 most active strains of lactobacilli were selected for development of 10 variants of probiotic consortia. Also, there was increase of adhesive activity in the consortia compared to other components. These consortia can be used for the treatment of BV in addition to metronidazole.

**Conclusion:**

The probiotic consortia identified in this study had high antagonistic, adhesive properties, and resistance to metronidazole. These probiotics can potentially be used for the development of biological products for the treatment and prevention of bacterial vaginosis.

